# Primary Ewing Sarcoma/Primitive Neuroectodermal Tumor of the Kidney: The MD Anderson Cancer Center Experience

**DOI:** 10.3390/cancers12102927

**Published:** 2020-10-11

**Authors:** Nidale Tarek, Rabih Said, Clark R. Andersen, Tina S. Suki, Jessica Foglesong, Cynthia E. Herzog, Nizar M. Tannir, Shreyaskumar Patel, Ravin Ratan, Joseph A. Ludwig, Najat C. Daw

**Affiliations:** 1 Department of Pediatrics, the University of Texas MD Anderson Cancer Center, Houston, TX 77030, USA; TSSuki@mdanderson.org (T.S.S.); jfoglesong@luriechildrens.org (J.F.); cherzog@mdanderson.org (C.E.H.); 2 Department of Pediatrics and Adolescent Medicine, American University of Beirut Medical Center, Beirut 1107, Lebanon; 3 Department of Investigational Cancer Therapeutics, the University of Texas MD Anderson Cancer Center, Houston, TX 77030, USA; rhsaid@stgeorgehospital.org; 4 Department of Biostatistics, the University of Texas MD Anderson Cancer Center, Houston, TX 77030, USA; CRAndersen@mdanderson.org; 5 Division of Hematology, Oncology, Neuro-Oncology & Stem Cell Transplant, Ann & Robert H. Lurie Children’s Hospital of Chicago, Chicago, IL 60611, USA; 6 Department of Genitourinary Medical Oncology, the University of Texas MD Anderson Cancer Center, Houston, TX 77030, USA; ntannir@mdanderson.org; 7 Department of Sarcoma Medical Oncology, the University of Texas MD Anderson Cancer Center, Houston, TX 77030, USA; spatel@mdanderson.org (S.P.); RRatan@mdanderson.org (R.R.); jaludwig@mdanderson.org (J.A.L.)

**Keywords:** Ewing sarcoma, kidney, renal, tumor thrombus, treatment, chemotherapy, survival, outcome

## Abstract

**Simple Summary:**

The Ewing sarcoma family of tumors (ESFT)s rarely originate in the kidneys and their treatment is significantly different from the other common kidney tumors. The standard treatment for ESFT of other sites includes multi-agent chemotherapy and local control with surgery and or radiation therapy. Limited information exists on the clinical behavior and management of ESFTs of the kidney. This study aims to describe our experience with these rare tumors over a period of 23 years at MD Anderson Cancer Center to identify potential prognostic factors and help develop management guidelines. We found that the 4-year overall survival for patients without metastasis was 85% compared to 47% for patients with metastasis. Patients with tumors confined to the kidney treated with nephrectomy and adjuvant chemotherapy have favorable outcomes. Local tumor extension beyond the kidney, tumor thrombus, and distant metastasis are unfavorable factors that warrant intensification or novel approaches of therapy.

**Abstract:**

Limited information exists on the clinical behavior of the Ewing sarcoma family of tumors (ESFT) of the kidney. We reviewed the records of 30 patients (aged 8–69 years) with ESFT of the kidney seen at our institution between 1990 and 2013. We analyzed the event-free survival (EFS) and overall survival (OS) for associations with patient demographics, disease group, tumor size, tumor thrombus, and treatment. Six patients (20%) had tumors confined to the kidney (Group I), seven (23.3%) had local tumor extension beyond the kidney (Group II), and 17 (56.7%) had distant metastasis at diagnosis (Group III). Twenty-five (83.3%) patients underwent radical (19 upfront, five delayed) or partial (one upfront) nephrectomy, 25 (83.3%) chemotherapy and four (13.3%) radiotherapy. The 4-year EFS and OS were 43% (95% CI, 26–61%) and 63% (95% CI, 46–81%), respectively. EFS and OS were significantly associated with disease group and chemotherapy (*p* < 0.039). The presence of tumor thrombus in renal vein and/or inferior vena cava was associated with worse EFS (*p* = 0.053). Patients with disease confined to the kidney treated with nephrectomy and adjuvant chemotherapy have favorable outcomes. Local tumor extension beyond the kidney, tumor thrombus, and distant metastasis are unfavorable factors that warrant intensification or novel approaches of therapy.

## 1. Introduction

The Ewing sarcoma family of tumors (ESFT) represents a group of small round cell neoplasms including osseous and extra-osseous Ewing sarcoma (EWS), soft tissue primitive neuro-ectodermal tumors (PNET), and malignant small-cell tumor of the thoracopulmonary region (Askin’s tumor) [1]. These highly aggressive sarcomas are poorly differentiated and commonly arise from the axial and appendicular skeleton of teenagers and young adults, but they also develop from soft tissue [2]. Extra-osseous EWS develops primarily in the trunk and extremities; rare sites including the retroperitoneum, head and neck, orbit, parameningeal, and genitourinary including bladder and prostate, have been described [3].

ESFT of the kidney is a very rare entity representing less than 5% of renal tumors [4]. It was first reported in 1975 [5] and has a non-specific clinical presentation and non-diagnostic radiological findings. Because of its rarity, these tumors are easily mistaken for other more common tumors involving the kidney. Tissue diagnosis is usually confirmed by demonstrating positive CD99 expression by immune-histochemistry and the presence of a reciprocal translocation between the *EWS* gene on chromosome 22 and members of the ETS family, most commonly *FLI1* or *ERG*. The most common chromosomal translocation results in an oncogenic EWS-FLI1 fusion protein involved in tumorigenesis. The N-terminal *EWSR1* gene belongs to the FET family of RNA binding proteins, which includes FUS, EWS, and TAF15. Presence of the fusion protein has been shown to recruit the BAF chromatin-remodeling complex (also known as SWI/SNF) to DNA binding sites, which dysregulates hundreds of genes and ultimately drives tumorigenesis [6,7].

Primary renal ESFTs have been characterized by a high potential for rapid metastasis leading to death [8,9]. In addition, there is no consensus on the optimal management of these tumors due to the paucity of available data. The current therapeutic approach has been extrapolated from the experience of treating ESFT of other sites with multimodal therapy, including multi-agent chemotherapy with surgery and, or radiation therapy [9,10,11]. Most published case series have described the clinical and, or histopathological features of these tumors [8,11,12,13,14], but few studies have described the treatment and patient outcomes [9,15,16,17]. Previous reports indicated that most patients with ESFT of the kidney present with advanced disease and have a guarded prognosis [18,19].

The purpose of the current study is to describe the clinical characteristics, treatment, and outcomes of patients with ESFT of the kidney who were seen at our institution to identify potential prognostic factors and help develop management guidelines for these rare tumors. 

## 2. Results

### 2.1. Patient Characteristics

Of 910 patients with ESFT seen at our institution over a period of 23 years, 31 (3.4%) had primary ESFT arising within the kidney. One patient was excluded from the analyses due to initial misdiagnosis and treatment as a renal cell carcinoma. The clinical characteristics of the 30 patients included in this study are summarized in Table 1. The most common presenting symptoms were flank or abdominal pain (70%) and hematuria (36.7%). Eight (26.7%) patients presented with systemic symptoms such as fever or weight loss. Imaging studies at presentation identified a large renal mass in all patients. In the 29 patients for whom data was available, tumor extension to the renal vein was present in two patients, to the inferior vena cava (IVC) in six, and to the IVC and right atrium in one. The diagnosis was made by primary tumor resection in 19 (63.3%) patients and by biopsy in 11 (36.7%) patients. *EWS* gene rearrangement was found in 17 of 19 tested tumors (89.5%) by polymerase chain reaction or fluorescent in situ hybridization analysis.

Six patients (20%) had tumor confined to the kidney (Group I), seven patients (23.3%) had local tumor extension beyond the kidney (Group II), and 17 patients (56.7%) had metastatic disease at diagnosis (Group III). The most common sites of metastasis were the lungs (*n* = 8, 47%) and bones (*n* = 6, 35.3%). Metastasis to the liver (*n* = 2), bone marrow (*n* = 2) and leptomeninges (*n* = 1) was also seen. The age distribution was similar among the three groups. The median largest tumor dimension was 11 cm (range, 4–19 cm). 

### 2.2. Treatment

#### 2.2.1. Surgery

All but one of the 13 (92.3%) patients in group I and group II underwent upfront nephrectomy (*n* = 11) or partial nephrectomy (*n* = 1); one patient in group II with tumor extension into the right atrium underwent a tumor biopsy followed by neoadjuvant chemotherapy and radical nephrectomy (patient #11). Of the 17 patients with metastatic disease at diagnosis, 10 (58.8%) patients had a tumor biopsy initially, and seven (41.2%) had upfront nephrectomy. Of the ten patients who had a biopsy initially, one patient had upfront nephrectomy followed by chemotherapy, and nine received neoadjuvant chemotherapy with delayed nephrectomy in four. Four patients did not undergo surgery because of disease progression while receiving chemotherapy (*n* = 3) and achieving complete remission with chemotherapy alone (*n* = 1), and data was not available in one patient (*n* = 1).

#### 2.2.2. Chemotherapy

Various chemotherapeutic agents and regimens were used and are summarized in Table 2. Of the 29 patients on whom data is available, four patients did not receive chemotherapy or received only one chemotherapy cycle due to patient refusal (*n* = 2), delay in establishing the diagnosis (*n* = 1), or unknown reason (*n* = 1). The most commonly used chemotherapeutic agents were vincristine, cyclophosphamide, doxorubicin, ifosfamide, etoposide, cisplatin, irinotecan, and temozolomide. The most common initial chemotherapy used was vincristine, doxorubicin, and cyclophosphamide (VDC) either alone (*n* = 7, 23.3%) or in combination with ifosfamide and etoposide (IE) (*n* = 7, 23.3%). The second most common combination used was vincristine, doxorubicin, and ifosfamide (VDI).

Of the six group I patients, four patients received adjuvant chemotherapy: VDC alone (*n* = 1), VDC alternating with IE (*n* = 1), VDC with VDI (*n* = 1), or ifosfamide and doxorubicin (ID) alternating with cisplatin and etoposide (PE) (*n* = 1). The remaining two patients were non-compliant and received only one cycle of chemotherapy (patient #2, 6).

Of the seven group II patients, five patients received adjuvant chemotherapy, and one patient with tumor extending through the IVC to the right atrium underwent an initial biopsy followed by neoadjuvant chemotherapy and delayed nephrectomy. These patients received the following combinations: VDC (*n* = 2), VDC alternating with IE (*n* = 2), VDI (*n* = 1) or other (*n* = 1). Two patients also received maintenance chemotherapy with irinotecan and temozolomide. The seventh patient with a tumor thrombus in the renal vein (patient #13) did not receive any chemotherapy as part of his primary treatment.

Of the 17 group III patients, 15 patients received chemotherapy, including VDC (*n* = 4), VDC alternating with IE (*n* = 4) or with PE (*n* = 1), VDI alone (*n* = 1) or combined with PE (*n* = 2), and other combinations (*n* = 3). One patient received only one cycle of chemotherapy (patient #27) and data was not available in another patient (patient #24).

#### 2.2.3. Radiation Therapy

Radiotherapy was used in a small number of patients: none in group I, one patient in group II (patient #11) received 45Gy to the tumor bed, and three patients in group III received radiation to the tumor bed (*n* = 2; patients #15, 16) and metastatic bone lesions (*n* = 3; patients #14, 15, and 16). 

### 2.3. Patient Outcomes

**Figure 1:**
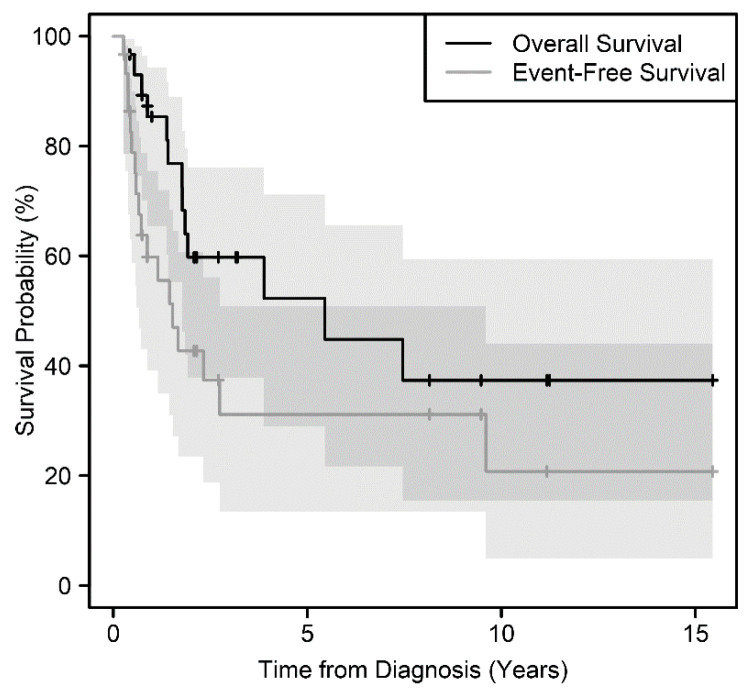
Overall survival and event-free survival distributions for 30 patients with ESFTs of the kidney. Shaded regions are 95% confidence intervals with darker shading where these overlap.

Of the 30 patients, 13 (43%) died, and 18 (60%) experienced relapse, progression, or death. Median event-free survival (EFS) and overall survival (OS) were 1.5 years and 5.5 years, respectively, with median follow-ups of 0.9 years and 1.9 years, over a range of 0.3 years to 15.4 years (Figure 1). The 4-year rates of EFS and OS for the 30 patients were 43% (95% CI, 26–61%) and 63% (95% CI, 46–81%), respectively. The 4-year rates of EFS and OS for patients without metastasis at diagnosis were 54% (95% CI, 27–81%) and 85% (95% CI, 65–100%), respectively, compared to those with metastasis 35% (95% CI, 13–58%) and 47% (95% CI, 23–71%), respectively.

Of the six patients with disease confined to the kidney (group I), four patients survived without evidence of disease. The remaining two patients, both of whom were not compliant with adjuvant chemotherapy, developed local recurrence: one had a short follow up of 11 months, and the other eventually died of metastatic disease. 

Of the seven patients with local tumor extension beyond the kidney (group II), four patients survived. None of these four patients had tumor extension to the renal vein or IVC, and all four underwent primary radical nephrectomy followed by adjuvant chemotherapy. Two of these four patients survived without evidence of disease, and the other two patients were alive with disease, one of whom had a late relapse at local and distant sites, and the second had a local relapse followed by pulmonary relapse. The remaining three patients died of disease; all three patients had a tumor thrombus at diagnosis. One of these three patients underwent primary radical nephrectomy and caval thrombectomy followed by adjuvant chemotherapy, then developed local relapse followed by pulmonary relapse. The second patient underwent upfront nephrectomy without adjuvant chemotherapy, then developed lung metastasis. The third patient underwent an initial biopsy followed by neoadjuvant chemotherapy, secondary radical nephrectomy and thrombectomy followed by radiation therapy 45Gy, then developed lung metastasis.

Of the 17 patients with metastatic disease (group III), all but one patient (94.1%) had tumor recurrence or progression on therapy. Three patients in this group (patients #19, 23, and 24) who started chemotherapy a median of 8 weeks (range, 7–9 weeks) after initial nephrectomy developed lung nodules after upfront tumor resection and before initiation of chemotherapy. Two of these three patients had a tumor thrombus extending through the IVC, and one had a pulmonary embolus after surgery without evidence of distant venous thrombus.

### 2.4. Prognostic Factors

All four patients with tumors confined to the kidney (Group I) who underwent radical nephrectomy followed by adjuvant chemotherapy were alive and disease-free. Three of 5 patients with local tumor extension beyond the kidney without distant metastasis (Group II) who underwent initial nephrectomy followed by adjuvant chemotherapy developed local and distant relapse and one patient (with a tumor thrombus extending to the right atrium) who received neoadjuvant chemotherapy, delayed nephrectomy and radiation to the tumor bed suffered a distant relapse. The presence of renal vein or IVC thrombus may contribute to the development of early lung metastasis. Three of five patients with a tumor thrombus in the renal vein or IVC and no metastasis at diagnosis developed metastasis to the lung within 2.5 months after nephrectomy and before initiating chemotherapy. Metastatic disease at diagnosis was associated with an adverse outcome; all but one patient with metastasis died of disease.

**Figure 2:**
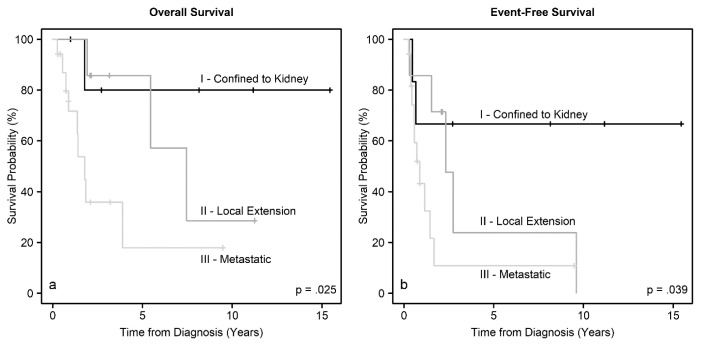
Overall survival and event-free survival distributions according to the disease group.

**Figure 3:**
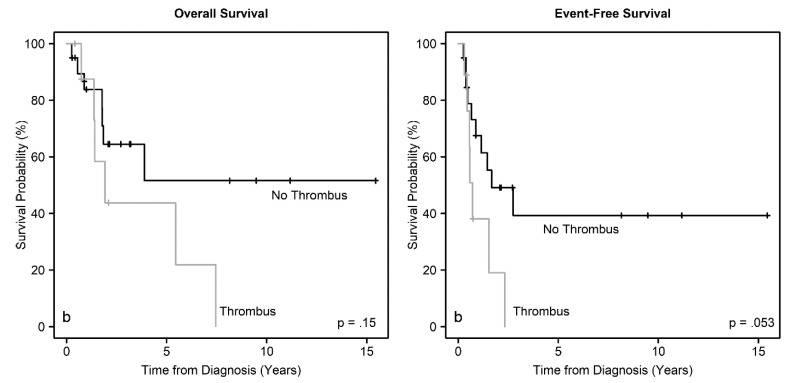
Overall survival and event-free survival distributions according to the presence of thrombus in the renal vein and, or IVC.

**Figure 4:**
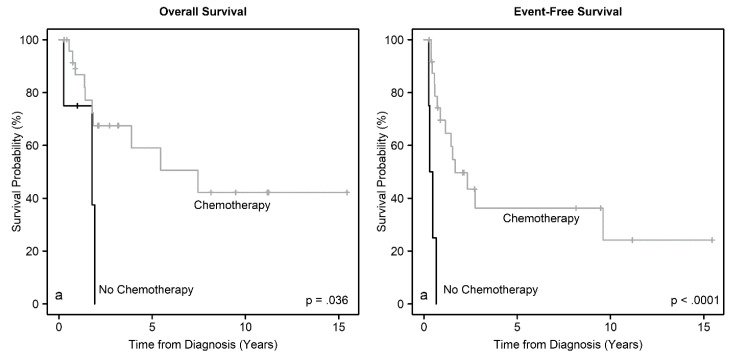
Overall survival and event-free survival distributions according to the use of chemotherapy.

We analyzed EFS and OS for associations with patient demographics, tumor size, disease group, presence of metastasis, tumor thrombus, and treatment. There were no significant associations between EFS or OS with age, sex, race, or ethnicity. There was a trend for worse EFS for patients with larger tumors (*p* = 0.07), but not with OS (*p* = 0.13). Importantly, we found significant associations between EFS (*p* = 0.039) and OS (*p* = 0.025) with disease group (Figure 2). As expected, the presence of metastasis was associated with worse EFS (*p* = 0.058) and OS (*p* = 0.029). Also, the presence of thrombus in the renal vein and, or the IVC was associated with worse EFS (*p* = 0.053), but the association with OS did not reach statistical significance (*p* = 0.15) (Figure 3). Furthermore, EFS (*p* < 0.0001) and OS (*p* = 0.036) were significantly associated with the use of chemotherapy (Figure 4), but not with whether the patient had surgery or not (*p* > 0.16). Timing of nephrectomy (upfront vs. delayed) was significantly associated with OS; 6 of 20 patients (30%) who underwent upfront nephrectomy died, whereas 4 of 5 patients (80%) who underwent delayed surgery died (*p* = 0.041). The association with EFS did not reach statistical significance (*p* = 0.20). 

## 3. Discussion

In this study, we reviewed our experience with 30 patients with ESFT of the kidney who were seen at our institution over a period of 23 years. We found that 20% of the patients had tumors confined to the kidney, 23.3% had tumors extending locally beyond the kidney (capsular invasion, perinephric tissue invasion, lymphovascular invasion, presence of renal vein and, or IVC thrombus, or extension within the abdomen excluding the liver), and 56.7% had metastasis at diagnosis. The 4-year rates of EFS and OS for patients without metastasis at diagnosis were 54% (95% CI, 27–81%) and 85% (95% CI, 65–100%), respectively, compared to those with metastasis 35% (95% CI, 13–58%) and 47% (95% CI, 23–71%), respectively. Patient outcomes were significantly associated with disease group, presence of metastasis, presence of tumor thrombus, and the use of chemotherapy.

Of all patients with ESFT seen at our institution, 3.4% had their tumors originating from the kidney. This rate is higher than the 1.5% rate reported by Zollner et al. [17], which could reflect the referral pattern to our institution and, or a prior underestimation of the true incidence of these tumors. In fact, reported cases of primary renal ESFT have been more frequent in recent years, mainly due to improved molecular studies, which allowed a more accurate diagnosis of renal tumors. The diagnosis of primary renal ESFT can be challenging. In addition to the morphological and immunohistochemical features, the diagnosis requires confirmation by identifying the pathognomonic genetic alteration. The 2020 WHO classification of soft tissue and bone tumours defines Ewing sarcoma as a small round cell sarcoma showing gene fusions involving one member of the FET family of genes (usually *EWSR1*) and a member of the ETS family of transcription factors [20]. In our series, we observed a preponderance for male sex (66.7%) and young adults (median age at diagnosis 30.5 years) as previously described [10,14,21]. The age group is somewhat different from the age distribution of EWS in general, which is diagnosed at a younger age usually less than 20 years old [2,22]. Similar to previous reports [10,11,12,15,17,21,23], the presenting symptoms of primary renal ESFT are non-specific as other renal tumor malignancies and various benign conditions may have a similar presentation [24]. The median largest dimension of the tumor at diagnosis was 11 cm, with a trend for worse EFS for patients with larger tumors. Tumor size has been associated with an unfavorable outcome in ESFT [25,26]. 

Our data showed that 56.7% of patients presented with metastatic disease at diagnosis, mainly to the lungs, liver, and bones. Additionally, three patients developed metastasis to the lung soon after diagnosis and before initiation of systemic therapy. These results are in concordance with previous reports describing metastasis in 40−65% of newly-diagnosed patients with primary renal EWS/PNET [11,16,17,23]. The rate of metastatic disease at diagnosis is significantly higher than that reported for patients with EWS of all sites [2,25,26]. This high incidence of metastatic disease reflects the aggressive nature of this tumor or a delay in diagnosis since the retroperitoneum is an ample space that allows growth of the tumor before manifestation of symptoms. 

The diagnosis of primary renal ESFT is challenging due to delayed symptoms, non-specific clinical presentation, and non-distinctive radiological features [11,12,21,23]. In fact, Lee et al. showed that the most common and representative imaging elements were the presence of a large necrotic and hemorrhagic mass with extensive venous thrombosis, similar to advanced renal cell carcinoma [27]. Other investigators suggested that some features may indicate a PNET rather than a renal cell carcinoma [27,28,29,30,31] including (1) the presence of bulky disease, (2) an endophytic infiltrative growth pattern, (3) the presence of multiple irregular septum-like structures, (4) the difference in the pattern of necrosis and hemorrhage within the mass that tends to be multifocal and diffuse in PNET and central in renal cell carcinoma, (5) the very weak enhancement of the tumor compared to the normal renal cortex and (6) a higher rate of renal vein thrombosis and distant metastasis. Further radiological studies are warranted to elucidate potential radiological characteristic features.

Our study showed that patients with disease confined to the kidney who underwent a complete resection followed by adjuvant chemotherapy had a favorable outcome, contrary to patients who did not complete recommended adjuvant treatment. We also observed that patients with local extension beyond the kidney responded initially to surgery and adjuvant chemotherapy, but had a significant rate of relapse (mainly local failure followed by distant metastasis) and that the presence of renal vein or IVC thrombus could contribute to the development of early pulmonary metastasis. Additionally, this study showed that despite several patients with metastatic disease having had an initial response to systemic chemotherapy, all but one of the 17 patients died of disease. Likewise, reported cases in the literature showed a high risk for recurrence/metastasis in patients with localized disease treated with nephrectomy alone [16,32,33] and poor outcomes in patients with metastatic disease [9,23]. Similar to ESFTs of all sites, the outcome of our patients with metastatic tumors of the kidney was dismal despite multimodal therapy, highlighting the need for novel treatment approaches for this very high-risk group.

Our findings suggest that surgery with adjuvant chemotherapy for patients with tumor confined to the kidney is potentially curative. Our results also affirm the importance of early initiation of chemotherapy before the development of metastasis and suggest a potential role for radiation therapy in local control. The standard of care for patients with ESFTs of all sites consists of neoadjuvant chemotherapy followed by local control (surgery and, or radiation therapy) and further chemotherapy. Our patients with primary ESFTs of the kidney who underwent upfront nephrectomy did not appear to have had an adverse survival outcome. In fact, 12 of our 13 patients with non-metastatic disease had upfront surgery and had a favorable outcome when adjuvant chemotherapy was used. Our finding of a worse survival for patients who underwent delayed nephrectomy likely reflects the advanced disease stage in these patients rather than a delay in local control. For Ewing sarcoma of all sites, radiotherapy is currently recommended for unresectable tumors or unexpected positive margins [34] and may play a role in the local control of primary renal tumors with local extension beyond the kidney [10]. Our patient with group II disease who received radiotherapy to the tumor bed did not have a local relapse but a distant relapse. Among patients with primary renal Ewing sarcoma treated according to the EURO.EWING.99 protocol, patients with locally advanced disease had local disease control and improved outcome when they received radiotherapy to the tumor bed [17]. A larger study of patients of primary EFTS of the kidney with similar disease extent treated in a homogenous fashion is needed to determine the optimal timing (upfront vs. delayed) of surgery and the role of radiation therapy in local control.

Three of our five patients with non-metastatic tumors and a tumor thrombus in the renal vein or IVC developed metastasis to the lung within 2.5 months after nephrectomy, suggesting a potential benefit from using neoadjuvant chemotherapy or initiation of systemic therapy soon after tumor resection [35]. The current standard approach to the most common primary renal tumors, renal cell carcinoma (RCC) in adults and Wilms tumor in children, in North America is upfront surgery whenever feasible. A renal mass biopsy may be obtained in selected patients such as those with unresectable or metastatic tumors as it allows early initiation of neoadjuvant chemotherapy and potentially improve tumor resectability. On the other hand, a renal biopsy is highly recommended in cases where the diagnosis of RCC or Wilms tumor is doubtful based on imaging findings. If the diagnosis of primary EFTS of the kidney is established by biopsy, administration of neoadjuvant chemotherapy may prevent the development of metastatic disease and offers the advantage of administering ifosfamide, a potentially nephrotoxic drug, prior to surgical consolidation. Although the risks previously associated with biopsy of renal tumors, including tumor dissemination along the track and concerns for inaccuracy in diagnosis, have become rare [28,29,30], performing a renal tumor biopsy at diagnosis is usually avoided. In all 11 patients from our cohort who underwent a percutaneous biopsy, no complications were reported, and the specimen obtained was adequate for an accurate immunohistological diagnosis and *EWS* gene rearrangement testing (7 of 9 tested positive). Successful percutaneous biopsies have been reported in the literature to diagnose primary renal ESFT [16,35,36,37] and should be considered in selected patients. 

Our study was limited by its retrospective nature, the small number of patients, the unavailability of complete data for some patients, the heterogeneity of the treatments received over the prolonged study duration, and the short duration of follow up for certain patients. In addition, molecular testing by either fluorescent in situ hybridization (FISH) or polymerase chain reaction (PCR) was not commonly performed in the 1990’s to confirm the diagnosis of ESFT [38] and hence not required for inclusion in this study, but it is currently routinely performed for proper characterization of ESFTs. The small number of patients in our study may not have permitted the detection of significant differences among variables and limited our ability to make definitive conclusions. A larger study of patients with molecularly confirmed ESFTs who are treated homogenously is needed to make firm conclusions about impact of therapy and survival outcomes. The rarity of primary renal EWS makes a prospective study not feasible. 

## 4. Materials and Methods

We searched the tumor registry database at MD Anderson Cancer Center to identify all patients with histologically proven primary renal ESFT who were diagnosed between January 1990 and December 2013. The diagnosis of EWS/PNET was based on histology, and the morphological appearance of the tumor combined with immunohistochemistry. Data on molecular testing of the tumor was collected when available, but confirmation by molecular testing was not required for study inclusion. Information regarding the clinical characteristics, treatment, and outcome of patients was collected by reviewing the medical records. All available reports on pathology findings and imaging studies were reviewed. Tumor size was obtained from measurements of pathological specimens from pathology reports for patients who underwent upfront nephrectomy or from reports of initial imaging studies for patients who did not. This retrospective review was approved by the Institutional Review Board at MD Anderson Cancer Center (PA13-0760).

### 4.1. Patient Classification

Patients were classified into three categories according to disease extension at diagnosis: (a) Group I was defined as a tumor confined to the kidney that was completely resected with negative resection margins, (b) Group II was defined as a tumor that extended locally beyond the kidney without distant metastasis with one of the following: (1) capsular invasion, (2) perinephric tissue invasion, (3) lymphovascular invasion, (4) presence of renal vein and, or IVC thrombus, or (5) tumor extension within the abdomen excluding the liver, (c) Group III was defined as the presence of distant metastatic disease.

### 4.2. Statistical Methods 

The EFS and OS were calculated from the time of diagnosis. For EFS, an event was defined as relapse/progression, second malignant neoplasm, or death from any cause, whichever occurred first. Local failure was defined as relapse in the operative bed, abdomen outside the operative bed, or pelvis; tumor spread to the liver was considered distant metastasis. OS and EFS were estimated using Kaplan-Meier plots [39], together with corresponding estimates of the median survival, and the median and range of follow-up time. The 4-year EFS and OS were estimated as percentages with 95% Wald confidence intervals.

Associations between EFS or OS and discrete variables including age, sex, race, ethnicity, tumor size, disease group, presence of metastasis, tumor thrombus, surgery, and chemotherapy were summarized by Kaplan-Meier models and assessed by the log-rank test. In the analysis of outcome survival in relation to chemotherapy use, patients who received only one cycle of chemotherapy were considered as not having received chemotherapy. In cases where the discrete variable had more than two levels, pairwise comparisons by the log-rank test were performed, and Hommel-adjusted *p*-values were reported to compensate for multiple comparisons. The Cox proportional hazard model was used to assess associations between EFS or OS and continuous variables (age and tumor size); a penalized spline was used to ensure proportionality of hazards in the models with relation to tumor size. A 5% level of statistical significance was assumed in all analyses. All statistical analyses were performed using R statistical software (version 3.6.3, R Core Team 2020).

## 5. Conclusions

Primary renal ESFT is a rare presentation of extraosseous EWS that may affect young adults and should be included in the differential diagnosis of primary renal tumors. Local tumor extension beyond the kidney, tumor thrombus and distant metastasis are unfavorable prognostic factors. The mortality rate is high for patients with locally advanced and metastatic disease despite multimodal therapy approaches. In patients with localized disease, complete surgical resection and adjuvant chemotherapy could provide long-term disease-free survival. Local tumor extension beyond the kidney is associated with a high risk of local and, or distant relapse, suggesting the need for further intensification of therapy. Confirmation of our findings in a larger cohort of patients with established molecular diagnosis treated in a homogenous fashion is needed.

## Figures and Tables

**Table 1:** Clinical Characteristics of 30 Patients with Primary ESFT of the Kidney.

Characteristic	No. (%)
Age at diagnosis, years	
Median	30.5
Range	(8−69)
Sex	
Male	20 (66.7)
Female	10 (33.3)
Race	
White	29 (96.7)
Asian	1 (3.3)
Ethnicity	
Non-Hispanic	19 (65.5)
Hispanic	9 (31)
Other	1 (3.4)
Group	
I	6 (20)
II	7 (23.3)
III	17 (56.7)
Tumor size, cm	
Median	11
Range	(4−19)
Thrombus in renal vein or inferior vena cava *	
Yes	9 (31)
No	20 (69)
Treatment ^	
Upfront radical nephrectomy	19 (65.5)
Delayed radical nephrectomy	5 (17.2)
Upfront partial nephrectomy	1 (3.4)
Chemotherapy	25 (86.2)
Radiation therapy	4 (13.3)

* Data not available for one patient; ^ Data not available about surgery for one patient and chemotherapy in another patient.

**Table 2:** Clinical characteristics, treatment and outcomes of 30 patients with primary ESFT of the kidney.

Patient	Age (Years)	Ethnicity/Sex	EWS Rearrangement (PCR and/or FISH)	Tumor Extent	Nephrectomy	Primary Treatment Chemotherapy, Radiation Therapy	Site of First Relapse, Time from Diagnosis	Patient Outcome
Group I patients: Tumor confined to the kidney								
1	14	H/M	+	None	Upfront	VDC, IE × 14 cycles, No RT		Alive, NED, 32 mo
2	21	W/M	+	None	Upfront	VDC × 1 cycle Non compliance, No RT	Local, 6 mo lung, 12 mo	Dead, 21 mo
3	24	W/F	D/U	None	Upfront	VDC × 4 cycles VDI × 2 cycles, No RT		Alive, NED, 185 mo
4	27	W/F	+	None	Upfront	VDC, No RT		Alive, NED, 97 mo
5	31	As/F	D/U	None on surgical specimen (Questionable RV and IVC thrombus on imaging)	Upfront (Preoperative embolization)	ID, PE × 15 weeks No RT		Alive, NED, 132 mo
6	41	H/M	+	None	Upfront (partial nephrectomy)	VDCA × 1 cycle Non compliance, No RT	Local recurrence, 9 mo	AWD, 11 mo
Group II patients: Tumor extending beyond the kidney								
7	18	W/M	+	D/U	Upfront	VDC, IE × 1 yr, No RT	Retroperitoneal, lungs, bone, 9 yrs	AWD, 134 mo
8	23	W/M	+	Perinephric fat, LV invasion adrenal gland, tail of pancreas	Upfront	VDI × 1 cycle, VAC × 3 cycles VD × 2 cycles IrT × 8 cycles, No RT		Alive, NED, 25 mo
9	32	W/M	D/U	Perinephric fat, LV invasion	Upfront	VDC × 6 cycles No RT	Local, 33 mo lungs, 38 mo	AWD, 37 mo
10	34	H/F	+	Perinephric fat	Upfront	VDI × 6 cycles, IrT × 8 cycles, No RT		Alive, NED, 25 mo
11	12	W/M	- (FISH and PCR)	Perinephric fat, LNs, RV and IVC thrombus (RA thrombus on imaging)	Delayed (following 15 weeks)	VDC, IE × 45 weeks RT, tumor bed, 45Gy	Lungs, 17 mo	Dead, 65 mo
12	45	W/M	D/U	Perinephric fat, LNs, RV and IVC thrombus	Upfront	VDC × 4 cycles No RT	Local, 28 mo lungs, 5 yrs	Dead, 89 mo
13	69	W/F	D/U	RV thrombus	Upfront	None	Lungs, 4 mo	Dead, 22 mo
Group III patients: Metastatic disease								
14	8	W/M	+	Bone and BM	Upfront	VDC, IE RT, bone metastasis	D/U	Lost to follow up, AWD, 3 mo
15	9	H/F	+	LNs, Bone	Delayed	VDIE × 6 cycles VI × 2 cycles RT, tumor bed, 45Co-60 & bone metastasis, 55.8Co-60	D/U	AWD, 10 mo
16	18	Ar/F	+	Local invasion, LNs, bone and BM	Delayed (following6 cycles)	VDC, IE × 14 cycles RT, tumor bed, 50.4Gy & bone metastasis	Bone and BM, 20 mo	Dead, 46 mo
17	19	H/M	+	Lungs	No	VDI × 6 cycles PE × 3 cycles VIrT × 2 cycles, No RT	Local progression, 5 mo	Dead, 21 mo
18	22	H/M	+	Local invasion, LNs, bone	Delayed (following 4 cycles)	VDC × 2 cycle, PE × 3 cycles, No RT	Distant progression, bones, 4 mo	Dead, 10 mo
19	23	W/M	D/U	Perinephric fat (post-operative pulmonary embolism, followed by lung metastases)	Upfront	VDI × 6 cycles No RT	Lungs, 11 mo	Dead, 22 mo
20	25	H/M	D/U	RV and IVC thrombus, lungs	Upfront	VDC × 4 cycles No RT	Distant progression, lungs, 7 mo	Dead, 16 mo
21	30	W/M	D/U	RV thrombus, lungs	Upfront	VDC, No RT	D/U	AWD, 8 mo
22	32	W/F	- (PCR)	LNs, RV and IVC thrombus, lungs	Delayed (following 4 cycles)	PE × 2 cycles PE/Taxol × 3 cycles No RT	Disease progression, D/U, 7 mo	Dead, 16 mo
23	32	H/M	+	Perinephric fat, RV and IVC thrombus (Lung nodules identified prior to chemo, absent at initial imaging)	Upfront (preoperative embolization)	VDC × 8 cycles, VIrT × 6 cycles No RT	Lungs, 24 mo	AWD, 25 mo
24	33	W/F	+	RV and IVC thrombus (post-operative pulmonary embolism, followed by lung metastases)	Upfront	D/U	D/U	AWD, 4 mo
25	34	H/M	+	LNs, RV and IVC thrombus, retroperitoneum, liver	Upfront	ID × 3 cycles No RT	Local progression, 5 mo	Dead, 8 mo
26	35	W/M	D/U	Perinephric fat, LV invasion, lungs	Upfront	VDC, IE × 6 cycles Auto-stem cell transplant No RT	Lungs, 17 mo	AWD, 38 mo
27	39	W/F	+	LNs, bone	No	IrT × 1 cycle, RT (D/U)	Disease progression, D/U	Dead, 3 mo
28	41	W/M	D/U	Local invasion, LNs, bone and leptomeningeal spread	D/U	VDC × 5 cycles, IE × 2 cycles, No RT	D/U	AWD, 5 mo
29	43	W/M	+	LNs, retroperitoneum	No	VDC × 4 cycles, No RT	Local and distant progression, bones, 4 mo	Dead, 6 mo
30	50	W/M	D/U	LNs (above and below diaphragm), liver	No	PE, ID, × 6 cycles VDI × 3 cycles Oral E × 15 mo, No RT		Alive, NED, 113 mo

Abbreviations: A-Actinomycin D, As-Asian, Ar-Arabic, AWD-Alive with disease, BM-Bone marrow, C-Cyclophosphamide, D-Doxorubicin, D/U-Data unavailable, E-Etoposide, F-Female, H-Hispanic, I-Ifosfamide, Ir-Irinotecan, IVC-Inferior vena cava, M-Male, mo-month, LN-Lymph node, LV-Lymphovascular, NED-No evidence of disease, P-Cisplatin, RA-Right atrium, RT-Radiation therapy, RV-Renal vein, T-Temozolomide, V-Vincristine, W-White/Non-Hispanic, yrs-years.
